# A novel bead-based fluorescence immunoassay for aldosterone

**DOI:** 10.1016/S1674-8301(11)60028-6

**Published:** 2011-05

**Authors:** Min Sun, Chao Liu

**Affiliations:** Department of Endocrinology, the First Affiliated Hospital of Nanjing Medical University, 300 Guangzhou Road, Nanjing, Jiangsu 210029, China.

**Keywords:** aldosterone, immunoassay, bead, extraction, multiplex

## Abstract

Aldosterone quantification helps evaluate the rennin-angiotensin-aldosterone system. The new bead-based multiplex platform has not been applied in aldosterone detection to achieve simultaneous measurements of multiple hormones. A new sensitive competitive bead immunoassay based on Luminex technology for detecting aldosterone in small sample volumes was developed using two-antibody coupled beads and biotinylated aldosterone as tracer in combination with an extraction step. The assay was validated in human and mouse samples and exhibited a linear working range from 10 to 1,000 pg/mL. The assay was reproducible and precise with intra-assay coefficient of variations (CVs) from 6.0% to 11.2%, inter-assay CVs from 8.0% to 13.0% and good recovery [(90-110)%] and linearity [(89-107)%]. Excellent correlation was found between this new assay and the reference method (*r* = 0.96, *P* < 0.000,1). The successful establishment of this assay provides high possibility for carrying out bead-based multiplex assay measuring aldosterone and other parameters simultaneously in one 50 µL sample so that the efficiency can be improved and precious samples can be saved.

## INTRODUCTION

Accurate assessment of all hormones has always been the key point of clinical and experimental endocrinology. Among all those critical hormones, aldosterone plays a role in regulating sodium and potassium balance and blood pressure homeostasis[1]–[5]. Measuring aldosterone level helps evaluate the rennin-angiotensin-aldosterone system (RAAS), which could be abnormal in several diseases. Over the past decade, several immunoassays for measuring aldosterone in the blood, saliva and urine have been reported[6]–[11]. The commonly used radioimmunoassay (RIA) and enzyme-linked immunosorbent assay (ELISA) are high-throughput, but can only detect one analyte in each assay.

Recently developed multiplex technology based on flow cytometry and multi-analyte profiling (xMAP) technology of Luminex provides a novel platform for simultaneous measurement of several analytes in relatively small volumes[12]–[18]. Tiny carboxylated polystyrene microspheres (5.6 µm beads), which are initially dyed with two different infrared fluorophores, are used in this multiplex system. By varying the ratio of the two fluorophores, up to 100 sets of different color-coded beads can be distinguished when they pass through the machine one by one. Each bead set can be coupled to a different biological probe, which theoretically makes this system possible to detect simultaneously up to 100 different analytes in a single well. This unique characteristic benefits the research in which the specimen volume is limited and/or repeated analysis is required, for example, rodent studies, and screening trials.

However, very few competitive immunoassays utilizing bead-based multiplex system has been developed for detecting steroid hormones, partially because of the small molecular weight and low circulating levels of those hormones. The reporting signal of multiplex system is the median fluorescence intensity (MFI) of each bead detected, not the total fluorescence intensity of all the beads. This signal collecting method restricts possibility of high counts in the assay, especially when the analyte is small and the limited surface area cannot provide two binding sites. In this case, competitive immunoassay format is needed, which requires limited reagents in the assay instead of saturated reagents so that the unknown analyte and the tracer can compete for ligand. Taking together, aldosterone measurement using bead-based multiplex system faces challenges compared with multiplex assays for measuring other biological probes.

Here, we describe a bead-based competitive immunoassay for measuring aldosterone in small volumes of plasma after extraction, which has been validated in human plasma samples and been compared with reference assay.

## MATERIALS AND METHODS

### Reagents and materials

*N*-hydroxysulfosuccinimide (sulfo-NHS) and 1- ethyl-3-(3-dimethylaminopropyl) carbodiimide hydrochloride (EDC) were purchased from Pierce (Schwerte, Germany). Carboxylated polystyrene microspheres and Micro Bio-Spin™ 6 Tris chromatography columns were by Bio-Rad (Hercules, USA). SA-PE, steroids, organic and other materials were from Sigma-Aldrich (Taufkirchen, Germany). All purchased steroids were of analytical reagent grade or highest percent purity (>98%).

An affinity purified rabbit anti-mouse IgG from DAKO (Hamburg, Germany) was first coupled to beads to appropriately orientate the specific antibody. The monoclonal antibody (mAb) against aldosterone was prepared as previously described[19]. Biotinylated aldosterone was produced and purified as previously described[6],[20] and was used as tracer in the immunoassay.

### Bead coupling

Antibody coupled to carboxylated polystyrene beads was run through the Micro Bio-Spin 6 Tris chromatography to remove azide, TRIS, glycine, or other nitrogen-containing compounds and exchanged to PBS (pH 7.4) buffer. The concentration of the rabbit anti-mouse IgGs was determined by spectrophotometry. The covalent coupling of the prepared rabbit anti-mouse IgGs to microspheres was performed following the protocol recommended by Bio-Rad. The amount of antibody added was titrated. Thereafter, microspheres were incubated overnight with 500 µL anti-aldosterone antibodies (4°C, continuous shaking in the dark) after 3-fold washing with assay buffer. Titration experiments were also done to find the best concentration. On the following day, beads were washed twice with blocking buffer to remove any unbound antibody and then counted on the hemacytometer. After concentrations were adjusted to 5.5×10^3^/mL with blocking buffer, beads were stored at 4°C until further use.

### Bead-based competitive assay procedure

Organic solvent extraction [dichloromethane (DCM)/polyethylene glycol (PEG) 10000 (100 mg/L)] of human plasma (50 µL) was carried out as previously described[6]. After phase separation, the organic phase was transferred to glass tubes and evaporated to dryness under a nitrogen stream. Samples were reconstituted in a buffer (4.2 g/L NaHCO_3_, 0.5 g/L NaCl, 0.2 g/L K_2_CO_3_) before measurement. Concentrations obtained upon measurements were multiplied by four to compensate for the dilution factor. Calibrators were prepared from an ethanolic stock of aldosterone by serial dilutions. Five thousand coupled beads were added into each well of a 96-well multi-screen BV filter plate (Millipore, Schwalbach/Ts, Germany). All calibrators and samples were measured in duplicate. After washing (PBS, 0.05% Tween-20) twice, 50 µL calibrators or reconstituted samples were added into the corresponding wells. Subsequently, 100 µL diluted tracer were added and the plate was sealed with aluminum foil before an overnight incubation (4°C, continuous shaking). The incubation was terminated on the following day by washing three times. Fifty µL SA-PE (2 µg/mL) were added and incubated for 30 min on a shaker at room temperature. Finally, after washing three times, beads were re-suspended in 125 µL assay buffer and the whole plate was read on a Bio-Plex 200 (Bio-Rad, Munich, Germany). The specific MFI of the beads corresponds with the amount of biotinylated aldosterone bound to the beads. For standard curve fitting and subsequent calculation of the analyte concentrations, data were transferred to the Wiacalc software (Perkin-Elmer LAS, Jügesheim, Germany).

### Titration of antibodies and tracer

#### Titration of rabbit anti-mouse IgG (primary antibody)

A biotinylated goat anti-rabbit antibody (Acris, Herford, Germany) was incubated with beads coupled with different levels of rabbit anti-mouse IgG at room temperature for 10 min. After incubation with SA-PE, the MFI was measured to demonstrate the relative amount of antibody coupled to the beads.

#### Anti-aldosterone antibody (secondary antibody) and tracer titration

Calibrators for standard curve were incubated with two-antibody coupled beads and tracer to fulfill a complete assay. By comparing the displacement of standard curves formed by different concentrations of anti-aldosterone antibody and tracer, best concentrations for secondary antibody and tracer were chosen considering both the maximum MFI value and the sensitivity of the standard curve.

### Assay validation and comparison

#### Cross-reactivity

Specificity of the monoclonal antibody against aldosterone was tested for a wide spectrum of endogenous and synthetic steroids (at x-fold the endogenously found or administered concentrations).

#### Analytical sensitivity

By measuring the calibrator zero in 20 wells on the same plate, the concentration corresponding to the mean±SD of the respective MFI was calculated from its intercept with the displacement curve. This is defined as the analytical sensitivity or lower limit of detection (LLOD) of the assays.

#### Precision

Three different sample pools with aldosterone at low, medium and high levels were determined 20 times, respectively, on one plate. Then, the intra-assay coefficients of variation (CV) were calculated. Another three pools (also with low, medium and high aldosterone concentrations) were determined in 10 consecutive assays and the mean values of duplicate wells in each run were then calculated for the inter-assay CVs.

#### Recovery and linearity

Recovery was determined by mixing different calibrators with samples. The ratio of actually measured value to expected value was expressed as percentage. Linearity was determined in a spiked plasma sample, which was serially diluted in standard matrix and measured.

#### Comparison to established assays

An in-house time-resolved fluorescence immunoassay (TR-FIA) for aldosterone with the same extraction step[6] was used as reference assay.

### Statistical analysis

The correlation between the logarithm results measured by two different assays was analyzed using Pearson's correlation coefficient in SPSS (Version17.0).

## RESULTS

### Antibody titration

**Fig. 1:**
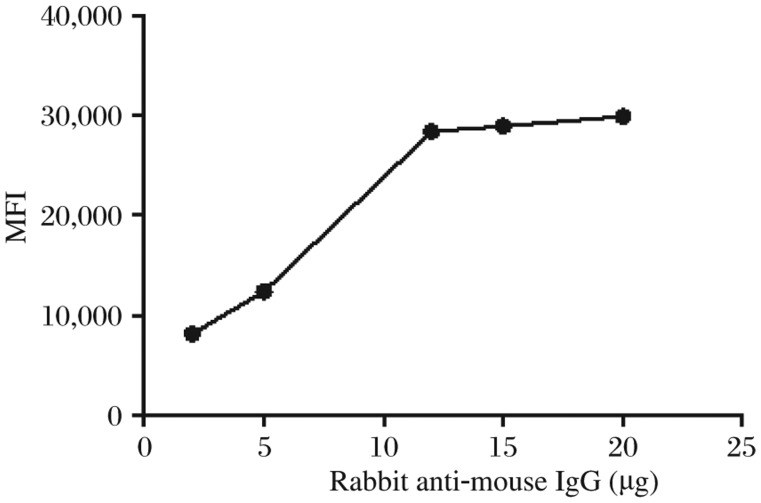
Titration of rabbit anti-mouse IgG coupled to beads. The median fuorescence intensity (MFI) generated by the biotinylated anti-rabbit IgG antibody was used to demonstrate the relative amount of the aimed antibody coupled to the beads. MFI increases with the amount of rabbit anti-mouse IgG added to each scale of beads and reaches the plateau when 12 µg is added.

**Fig. 2:**
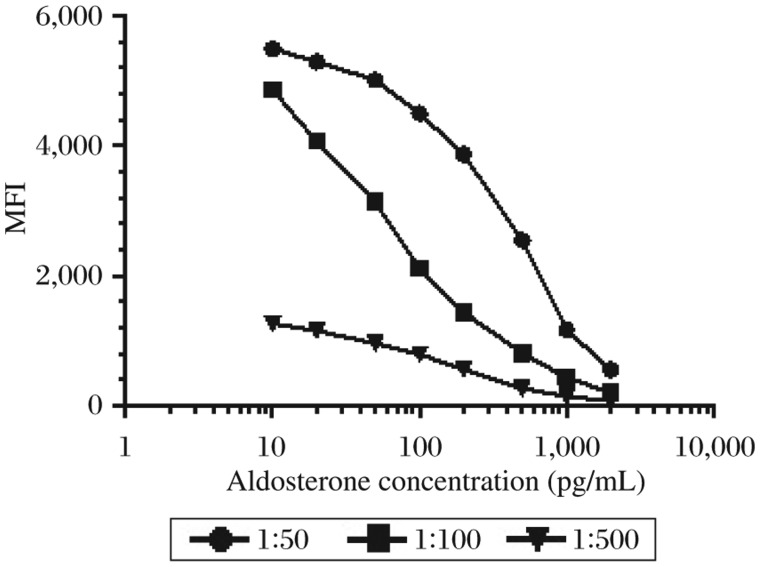
Titration of anti-aldosterone antibody. Standard curves for the displacement of aldosterone obtained by different titrations of anti-aldosterone antibody used for the second step of bead coupling. The curves show median fuorescence intensity (MFI) at maximal binding when no unlabeled aldosterone is present in the calibrators and decreasing counts with reduced binding due to increasing concentrations of unlabeled aldosterone competing for binding.

The amount of rabbit anti-mouse IgG added into each scale of beads was optimized by comparing the MFI obtained at different levels of the antibody. The MFI increased as more antibodies were present, but 12 µg was the saturated level for the beads (***Fig. 1***). The amount of the specific detecting antibody coupled to the beads was adjusted in order to give optimal sensitivity and high MFI as well. ***Fig. 2*** shows the different standard curves obtained with increasing dilutions of the anti-aldosterone antibody. Although the estimated doses were 50% maximal binding dose, ED50 was similar in the two lower curves. MFI dropped significantly at the titration of 1:500, and consequently 1:100 was chosen as the final coupling concentration.

### Tracer titration

**Fig. 3:**
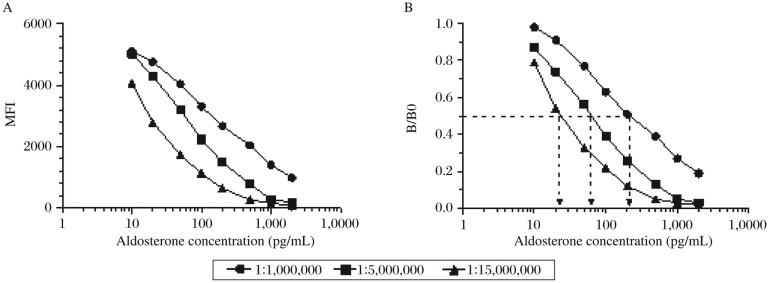
Titration of biotinylated aldosterone (tracer). Displacement of calibration curves obtained by different titrations of biotinylated aldosterone added into the assay. Y axis is median fuorescence intensity (MFI) (A) and B/B0 (B), respectively. B/B0 is the percentage of MFI at each point compared with that of calibrators zero. The arrows show the ED50 of each calibration curve.

Since this immunoassay design was based on competitive format, which emphasizes the limitation of reagents in assay, the best concentration of biotinylated aldosterone was selected to balance the higher MFI and the more sensitive standard curves. The MFI increased with the concentration of the tracer (***Fig. 3A***), but too much tracer in the system resulted in the loss of assay sensitivity (***Fig. 3B***).

### Selection of experimental conditions for antigen-antibody reaction

**Fig. 4:**
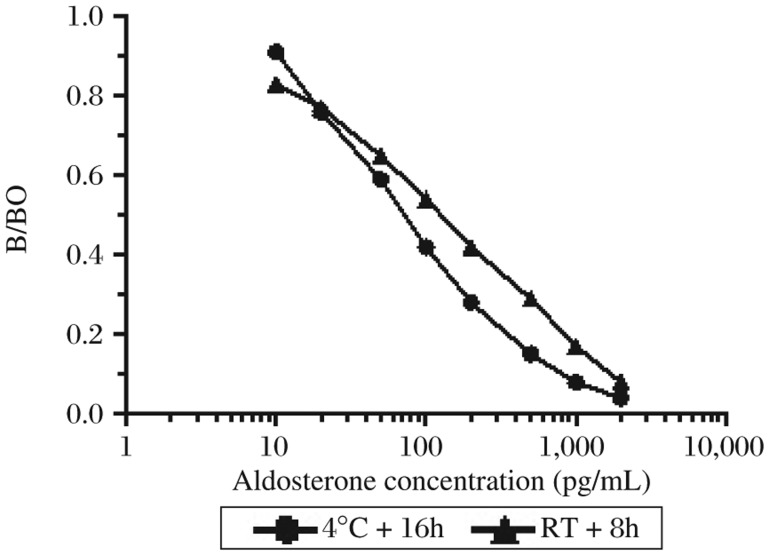
Impact of antigen and antibody incubation temperature and time on standard curves.

The MFI increased as the antigen-antibody reaction extended and reached plateau after 16 h at 4°C and 8 h at room temperature, respectively (data not shown). Comparing calibration curves, incubation for 16 h at 4°C yielded not only higher MFI, but also lower ED50 (***Fig. 4***). In the following, overnight (around 16 h) incubation at 4°C was affixed for the batch immunoassays.

### Specificity assessment

The cross-reactivity was calculated according to the following equation: Cross-reactivity% =Standard ED50/Cross-reactant ED50, and the results are summarized in ***Table 1***. Overall, the two highest cross-activities (0.083% and 0.089%) were observed for 18-oxocortisol and 18-hydroxycorticosterone, respectively. No significant cross-activity was found between the anti-aldosterone antibody and most of the endogenous and synthetic steroid tested.

**Table 1:** Cross-reactivities of anti-aldosterone antibody

Steroid	Cross-activity (%)
Aldosterone	100
18-oxocortisol	0.083
18-hy droxy corticosterone	0.089
Hydrocortisone	<0.0025
18-hydroxycortisol	<0.0025
Corticosterone	<0.00003
Deoxycorticosterone	<0.00003
Deoxycortisol	<0.00003
Progesterone	<0.00003
Hydroxycorticosterone	<0.00003
Dexamethasone	<0.00003
Estrone	<0.00003
Estradiol	<0.00003
Estriol	<0.00003
Testosterone	<0.00003

### Assay validation

The assay showed very excellent performance characteristics (***Table 2***). The LLOD was 9.8 pg/mL. For assay precision, the intra-assay CVs were from 6.0% to 11.2% and the inter-assay CVs were between 8.0% and 13.0%. The recovery and dilution linearity rates all fell within satisfactory ranges (90%-110%, 89%-107%, respectively).

**Table 2:** Summary of assay characteristics

Performance characteristics	Range
Variable	%CV (mean concentration)
LLOD	9.8 pg/mL
Linear	10-1,000 pg/mLcc
Precision intra-assay CV	11.2% (46 pg/mL)
	9.6% (405 pg/mL)
	6.0% (743 pg/mL)
Precision inter-assay CV	13.0% (52 pg/mL)
	11.2% (424 pg/mL)
	8.0% (786 pg/mL)
Recovery	90%-110% (26-1,026 pg/mL)
Dilution linearity	89%-107% (2 to date 32-fold dilution, 245-16 pg/mL)

LLOD: lower limit of detection.

### Comparison to in-house TR-FIA

**Fig. 5:**
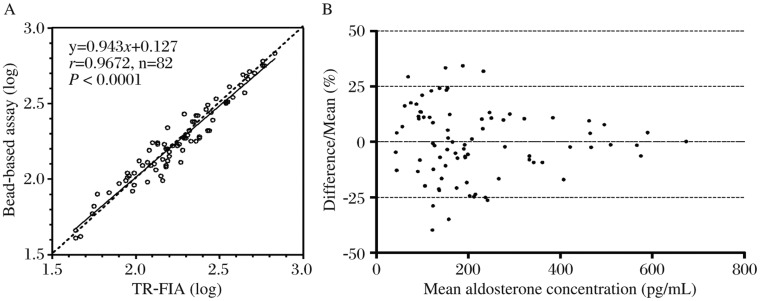
Correlation between bead-based assay and a TR-FIA. A: Comparison of aldosterone in 82 extracted human plasma samples measured by bead-based assay and the reference in-house TR-FIA. B: Bland-Altman plots of the same data.

Eighty-two human plasma sample values measured using our newly developed bead-based assay correlated well with those measured by the reference TR-FIA c(*y*=0.943*x*+0.127, *r*=0.9672, *n*=82, *P* < 0.0001, ***Fig 5A***). Bland-Altman plot analysis (***Fig. 5B***) showed that the results were scattered symmetrically around the zero line, indicating random deviations with no systematic difference.

## DISCUSSION

The potential role of excess levels of aldosterone in hypertension and metabolic syndrome has been highlighted in recent years[5],[21]–[27]. We aimed to develop a tool for assessment of aldosterone in small sample volumes utilizing the newly emerged bead-based multiplex system. So far, very few immunoassays have been reported for measurement of steroid hormones in bead-based system. Compared with peptide hormones, steroid hormones require a competitive assay format involving labeled hormone as a tracer. On the other hand, according to the principle of competitive immunoassay, the amount of the specific antibody binding sites and tracer should be limited. Lastly, as mentioned before, MFI is the median count instead of the total counts of the beads measured. Taken together, these will lead to problems in signal generation in bead-based multiplex system.

Among all the steroid hormones, aldosterone is the “minority hormone”, which circulates at very low concentration, with plasma cortisol concentration in human up to 2,000 times higher than aldosterone[28]; therefore, the measurement of aldosterone is often difficult and inconsistent in the levels reported so far. In the present study, we report the development of an immunoassay designed to detect levels of aldosterone in small volumes of human or mouse plasma samples with an extraction step in the bead-based multiplex system. It is well established that accurate detection of such low level steroids is better achieved after extraction[29],[30], a procedure not yet applied to bead-based assay. To our knowledge, our assay is the first one to measure aldosterone in bead-based system with an extraction step.

To simplify the bead coupling procedure, we first tried to couple the anti-aldosterone mAb directly to the beads, as all the reported bead-based assays do, but obtained very low counts even when the antibody added was at original concentrations (data not shown). The covalent coupling is between the carboxyl group on the surface of the polystyrene beads and the primary amines of antibody. The antigen-binding site of the specific antibody could be blocked during the covalent coupling. The two-step coupling as described in this paper using an antibody against mouse IgG as a bridge to connect the beads and the specific anti-aldosterone antibody can increase the MFI to display a satisfactory standard curve. One unblocked antigen-binding site of the primary antibody can catch several anti-aldosterone antibodies so that the signal is amplified by this means.

The optimal concentrations of both the primary and the secondary antibody are determined to balance the MFI count and the cost of the assay. As another part of the antigen-antibody compound generating fluorescent signals, the concentration of tracer, i.e. biotinylated aldosterone in our assay, also affects the MFI and sensitivity of the standard curves. So, the titration for the best concentration is necessary to optimize the assay.

All the endogenous and synthetic steroid hormones have similar structure, so the specificity of the detecting antibody is crucial for the accurate measurement. The monoclonal anti-aldosterone antibody used showed no cross-reaction with most of the steroids tested. The highest cross-reaction was found for 18-hydroxycorticosterone (0.089%); however, since the physiological level of 18-hydroxycorticosterone is not higher than aldosterone levels, this less than 0.1% cross-reaction can hardly affect the aldosterone detection. The very specific antibody in the present assay guarantees what is counted in the assay is exactly aldosterone.

The validation criteria for the assay have been met accordingly, and all the performance characteristics show that this is a sensitive, accurate and reproducible immunoassay with good linearity and recovery. One of the advantages worthy of mentioning is that the very small amount of only 50 µL plasma required for a duplicate measurement. This is especially useful for procedures requiring repeated blood drawing, for example, that recovered by tail vein bleeding. The linear range of this assay is large enough to cover both physiological and abnormal levels of aldosterone in human and rodents.

We compared values of aldosterone in human plasma samples measured by this new method with an in-house established immunofluorometric assay. The two assays showed overall significant correlation. To examine the bias further, we carried out Bland-Altman plot analysis. Symmetrically scattered points indicate that there is no obvious method bias but only random bias, and verify the excellent consistency between the two assays further.

In summary, a new immunoassay based on multiplex system has been established and validated for measuring aldosterone in small sample volumes. We demonstrated that competitive immunoassays for steroid hormones at very low circulating levels can be carried out on this platform in combination with extraction. The successful establishment of this assay provides very high possibility of simultaneous measurements of several analytes including steroid hormones in the same sample when it is combined with some other bead-based assays.
